# Genome-Wide Association Study Reveals the Genetic Basis of Five Quality Traits in Chinese Wheat

**DOI:** 10.3389/fpls.2022.835306

**Published:** 2022-03-03

**Authors:** Shuiyuan Hao, Hongyao Lou, Haiwei Wang, Jinghong Shi, Dan Liu, Baogerile, Jianguang Tao, Sanming Miao, Qunce Pei, Liangliang Yu, Min Wu, Ming Gao, Naihu Zhao, Jinchao Dong, Mingshan You, Mingming Xin

**Affiliations:** ^1^College of Agronomy, China Agricultural University, Beijing, China; ^2^Safety Production and Early Warning Control Laboratory of Green Agricultural Products in Hetao Region, Hetao College, Bayannur, China; ^3^Institute of Hybrid Wheat, Beijng Academy of Agriculture Forestry Sciences, Beijing, China; ^4^Department of Agriculture, Hetao College, Bayannur, China; ^5^Department of Medicine, Hetao College, Bayannur, China; ^6^Department of Library, Hetao College, Bayannur, China; ^7^Bayannur City Meteorological Bureau, Bayannur, China; ^8^Bureau of Agriculture and Animal Husbandry of Linhe District of Bayannur, Bayannur, China; ^9^Bureau of Agriculture and Animal Husbandry of Urat Middle Banner of Bayannur, Bayannur, China

**Keywords:** wheat, quality trait, GWAS, QTL, favorable alleles

## Abstract

Bread wheat is a highly adaptable food crop grown extensively around the world and its quality genetic improvement has received wide attention. In this study, the genetic loci associated with five quality traits including protein content (PC), gluten content (GC), baking value (BV), grain hardness (HA), and sedimentation value (SV) in a population of 253 Chinese wheat grown in Inner Mongolia were investigated through genome wide association mapping. A total of 103 QTL containing 556 SNPs were significantly related to the five quality traits based on the phenotypic data collected from three environments and BLUP data. Of these QTL, 32 QTL were continuously detected under at least two experiments. Some QTL such as *qBV3D.2/qHA3D.2* on 3D, *qPC5A.3/qGC5A* on 5A, *qBV5D/qHA5D* on 5D, *qBV6B.2/qHA6B.3* on 6B, and *qBV6D/qHA6D.1* on 6D were associated with multiple traits. In addition, distribution of favorable alleles of the stable QTL in the association panel and their effects on five quality traits were validated. Analysis of existing transcriptome data revealed that 34 genes were specifically highly expressed in grains during reproductive growth stages. The functions of these genes will be characterized in future experiments. This study provides novel insights into the genetic basis of quality traits in wheat.

## Introduction

Bread wheat (*Triticum aestivum* L.), which is a highly adaptable food crop cultivated globally, supplies approximately 20% of the total calories consumed by humans (Shiferaw et al., 2013; Shewry and Hey, 2015). As a staple food for about one-third of the global population, wheat is an important source of protein, vitamins, dietary fiber, and minerals. Because of its unique gluten properties, wheat can be used to produce various foods (Kumar et al., 2018). The improvement of living standards for many people worldwide and the development of the flour processing industry have forced breeders to focus on generating new high-quality wheat varieties (Curtis and Halford, 2014; Gao et al., 2021).

Wheat quality is a comprehensive trait that is influenced by the individual or combined effects of diverse traits, including grain protein content, gluten protein quality, grain hardness, flour color, starch quality, and nutrient element content; these traits are controlled by multiple quantitative trait loci (QTL) and are very sensitive to agro-environmental factors (Wang et al., 2018). Exploring the genetic basis of quality traits will accelerate the breeding of high-quality wheat varieties. Despite the complexity and limited genetic diversity of the wheat genome, there has been substantial molecular genetic research into wheat quality traits, which has resulted in the identification of some genes/QTL associated with quality traits through reverse genetics and traditional mapping methods (Uauy et al., 2006; He et al., 2007, 2009; Kumar et al., 2009; Crawford et al., 2011; Sun et al., 2016; Boehm et al., 2018; Jin et al., 2018; Goel et al., 2019; Semagn et al., 2021).

The traditional QTL mapping method usually involves the hybrid population of two parents and the detection of target trait loci via linkage mapping (Bettgenhaeuser and Krattinger, 2019). The limitation of this method is that relatively few recombination events occur during artificial hybridization, which leads to low resolution (Rabbi et al., 2021). Fine mapping requires the investment of considerable time, labor, and resources to develop many recombinant populations. Additionally, the evaluation of wheat quality traits necessitates the large-scale production of seeds to be ground into flour, which is one of challenges to traditional QTL mapping. With the rapid development of genome sequencing technology and high-density SNP arrays for genotyping (e.g., 9, 35, 55, 90, 660, and 820), the genome-wide association study (GWAS) has become an increasingly popular and promising alternative to linkage mapping for elucidating the genetic basis of complex traits, especially quality traits (Yu et al., 2006; Zhu et al., 2008; Ji et al., 2021). This method enables the use of natural populations without interbreeding to produce progeny, making it convenient for phenotype and genotype identifications. Thus, GWAS has been widely used for studying multiple traits in various plant species, such as Arabidopsis (Meijon et al., 2014; Proietti et al., 2018), rice (Yano et al., 2016; Tang et al., 2019), maize (Wang et al., 2016; Luo et al., 2019), and sorghum (Xie et al., 2019), as well as for investigating wheat agronomic traits (Liu et al., 2017; Sun et al., 2017) and disease resistance (Wu et al., 2021). The results of these studies provide useful information for identifying the genetic loci affecting wheat quality traits via GWAS.

In China, which is the largest wheat producer after the European Union, there are 10 wheat-growing regions (Sun et al., 2017). The wheat cultivated in the Hetao region of Inner Mongolia in the northern spring wheat region is well-known for its excellent quality, which is the result of the local climatic conditions (e.g., large day-to-night temperature changes, long-day conditions, and a long frost-free period). Improving wheat quality through molecular breeding will enhance the competitiveness of Hetao wheat in the international market. Furthermore, to the best of our knowledge, there have been no genetic studies on the Hetao wheat accessions conducted to identify and characterize the QTL associated with quality-related traits. Therefore, the present study was undertaken to identify genes/QTL related to wheat quality traits across different environments in Inner Mongolia using an association mapping approach combined with a high-density SNP marker assay.

## Materials and Methods

### Plant Materials and Experimental Design

The association panel used in this study consisted of 253 spring and semi-winter common wheat accessions collected from various wheat-producing regions in China, but mostly from the Inner Mongolia Autonomous Region and Sichuan province. These accessions were grown in Hangjihouqi (HJHQ; 40.85°N, 107.12°E), Linhe (LH; 40.73°N, 107.37°E), and Wulateqianqi (WLT; 40.73°N, 108.64°E) in the Inner Mongolia Autonomous Region during the 2019–2020 cropping season. Field trials were conducted using a randomized complete block design with three replications. Each accession was grown in a single row (2 m long) with 25 cm separating rows. Fertilizers were applied according to the following schedule: (1) 35 kg of diammonium phosphate and 10 kg of potassium nitrate were used as seed fertilizers (2) 40 kg of urea and 25 kg of compound fertilizer (15:15:15) were applied at the first and second watering, respectively. Other cultivation and management measures refer to local agronomic practices.

### Phenotypic Data Analysis

After the seeds harvested from the plants were dried and cleaned, the following five quality-related traits were analyzed by near-infrared spectroscopy using an Infraneo Senior instrument (Chopin Technologies, France): protein content (PC), gluten content (GC), baking value (BV), sedimentation value (SV), and grain hardness (HA). The near-infrared calibration model of wheat was developed and calibrated by the Application Department of Chopin Technologies, France, using the partial least squares method. Each sample was examined twice and the average value was used for further analyses. The R software package “pastecs” was used for the statistical analysis of phenotypic data and the analysis of variance for all five traits in different environments. The formula for the best linear unbiased estimate (BLUP) of phenotype is: *y* = *Xb* + *Zu* + *e*, where *y*, *b*, *u*, and *e* represent the observed phenotype, fixed effect vector, random effect vector, and residual, respectively, and *X* and *Z* represent incidence matrices. The R package “lme4” was used to estimate BLUP values for each line, considering environmental effects as fixed and genotype as random: *y* ∼ *(1 | rep% in% env)* + *(1 | env)* + *(1 | lines)* + *(1 | env: lines)*, where *rep% in% env* represents replications were nested within the environments. The broad-sense heritability (*h*^2^) of each trait was calculated as follows: *h^2^* = σ*^2^_*g*_/(*σ*^2^_*g*_* + σ*_*ge*_^2^/n* + σ*_*er*_^2^/nr)*, where σ*^2^_*g*_*, σ*_*ge*_^2^*, and σ*_*er*_^2^* represent the variance components for genotype, genotype × environment, and error, respectively, and *n* and *r* are the number of environments and replications, respectively. Correlations between traits were analyzed using the R package “corrplot.”

### SNP Genotyping

A wheat 55 K SNP array comprising 53,063 markers was used to genotype the association panel by China Golden Marker (Beijing) Biotech Co., Ltd^1^. The SNP data were analyzed using the Illumina GenomeStudio genotyping software. After filtering out the SNPs with a minor allele frequency (MAF) < 5% and a missing rate > 10%, the remaining 30,792 high-quality SNPs were included in the subsequent analyses.

### Population Structure, Principal Component, and Linkage Disequilibrium Analyses

The genetic structure of the natural population was assessed using two programs. First, the ADMIXTURE software (Alexander et al., 2009) was used to estimate the population structure of the panel according to polymorphic SNPs. The *K* value (number of hypothetical groups) ranged from 1 to 10, and the highest delta *K* value was set as the optimal value as previously described (Evanno et al., 2005). Second, the GCTA software (Yang et al., 2011) was used to perform a principal component analysis for the association panel. The R packages “Plot3D” and “Scatterplot3d” were used to prepare the figures presenting the results of the principal component analysis. The kinship matrix (K) was calculated using the Centered IBS method of the TASSEL software (Bradbury et al., 2007). LD analysis was performed using the TASSEL software. LD was estimated by pairwise comparisons among filtered SNP markers using squared allele frequency correlations (r2). The parameters for calculating r2 are to set “Select LD Type” to sliding window and “LD Window Size”to 1000. The LD decay distance was determined as the distance at which *r*^2^ decreased to half of the maximum value.

### Genome-Wide Association Analysis

A GWAS was conducted using the rMVP 1.0 software (Yin et al., 2021). More specifically, the General Linear Model (GLM), Mixed Linear Model (MLM), and Fixed and random model Circulating Probability Unification (FarmCPU) were used, of which MLM considered the population structure and relative kinship simultaneously. Marker–trait associations were identified on the basis of the polymorphic SNPs as well as the phenotypic data for the five wheat quality traits in different environments and the BLUP values. Considering the differences in the efficiency of GWAS for determining the marker–trait associations for different traits, two threshold P values were calculated according to the number of markers (P1 = 1/n for BV, GC, and PC; P2 = 0.05/n for HA and SV; n = total number of SNPs with MAF > 0.05). The R package “man” was used to prepare quantile–quantile plots and Manhattan plots.

### Putative Candidate Gene Analysis

To identify potential candidate genes, the IWGSC database^2^ was used to retrieve high-confidence genes located within 4 Mb of the LD region flanking significant SNPs included in stable QTL revealed by GWAS. Gene annotations and the homologs of specific wheat genes in rice and Arabidopsis were investigated using the Triticeae-Gene Tribe website^3^ (Chen et al., 2020). The reference genomes of the following species were used in this study: *T. aestivum* (IWGSC RefSeq v1.1), *Oryza sativa japonica* group (IRGSP 1.0), and Arabidopsis (TAIR10). The publicly available wheat transcriptome database was used to investigate the spatiotemporal expression signature of candidate genes, and transcripts per kilobase million (TPM) values were used to represent the expression levels of candidate genes. TPM values for candidate genes in five tissues (root, leaf, shoot, spike, and grain) and two growth stage (vegetative and reproductive) obtained from the wheat expression Database^4^ (Borrill et al., 2016; Ramírez-Gonzaìlez et al., 2018).

## Results

### Molecular Markers, Population Structure, Principal Component Analysis, and LD

A total of 30,729 high-quality SNP markers were identified across all 21 wheat chromosomes (Table 1 and Supplementary Figure 1), of which 10,879, 12,142, and 7,771 markers were located in the A, B, and D subgenomes, respectively. The total chromosome length spanned by these SNPs was 14039.31 Mb, with 6D and 3B having the shortest (473.52 Mb) and longest (829.73 Mb) chromosome length, respectively. The distance between SNPs ranged from 0.34 Mb on chromosome 1A to 0.65 Mb on chromosome 4D, with a genome-wide average of 0.46 Mb.

**TABLE 1:** SNP marker distribution in the A, B, D, and whole genomes.

Chr	No. of SNPs				Map length (Mb)				SNP density (Mb/SNP)			
				
	A	B	D	Total	A	B	D	Total	A	B	D	Total
1	1753	1729	1233	4715	593.25	688.70	495.13	1777.08	0.34	0.40	0.40	0.38
2	2011	1646	1280	4937	778.74	798.75	650.88	2228.37	0.39	0.49	0.51	0.45
3	1479	1761	1035	4275	749.87	829.73	615.46	2195.06	0.51	0.47	0.59	0.51
4	1138	1940	597	3675	743.32	673.47	509.18	1925.97	0.65	0.35	0.85	0.52
5	1877	1865	1116	4858	709.19	712.40	564.75	1986.34	0.38	0.38	0.51	0.41
6	1078	1563	1061	3702	616.94	720.49	473.52	1810.95	0.57	0.46	0.45	0.49
7	1543	1638	1449	4630	733.94	747.32	634.28	2115.54	0.48	0.46	0.44	0.46
All	10879	12142	7771	30792	4925.25	5170.86	3943.20	14039.31	0.45	0.43	0.51	0.46

**FIGURE 1:**
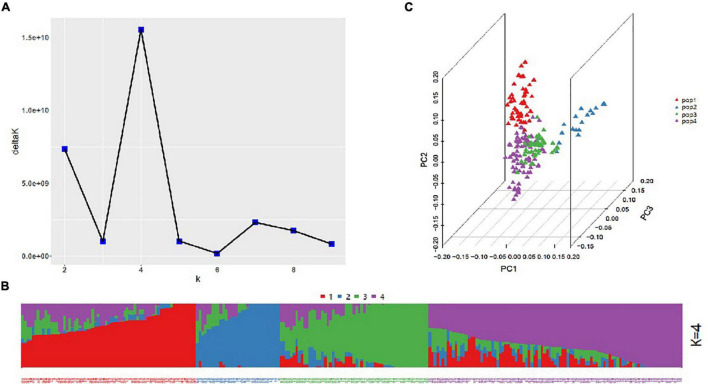
Population structure of the association panel. **(A)** Plot of delta *K* values vs. putative K values (1–10). **(B)** Stacked bar plot of the ancestral relationships among 253 accessions (*K* = 4). **(C)** Principal component analysis results.

The highest delta *K* value representing genetic clusters revealed by ADMIXTURE was calculated as 4 when 30,729 polymorphic markers were used; hence, the 253 accessions were divided into four subgroups (pop1, pop2, pop3, and pop4) (Figure 1), which was consistent with the result of principal components analysis based on the first three components contribution. Specifically, pop1, pop2, pop3, and pop4 comprised 67, 32, 57, and 97 accessions, respectively. The LD decay patterns varied by subgenomes. The LD decay distance was largest (approximately 7.6 Mb) and smallest (approximately 3.5 Mb) in the B and D subgenomes, respectively. The average LD decay distance for the whole genome was approximately 4 Mb (Supplementary Figure 2).

### Phenotypic Variations and Correlations

**FIGURE 2:**
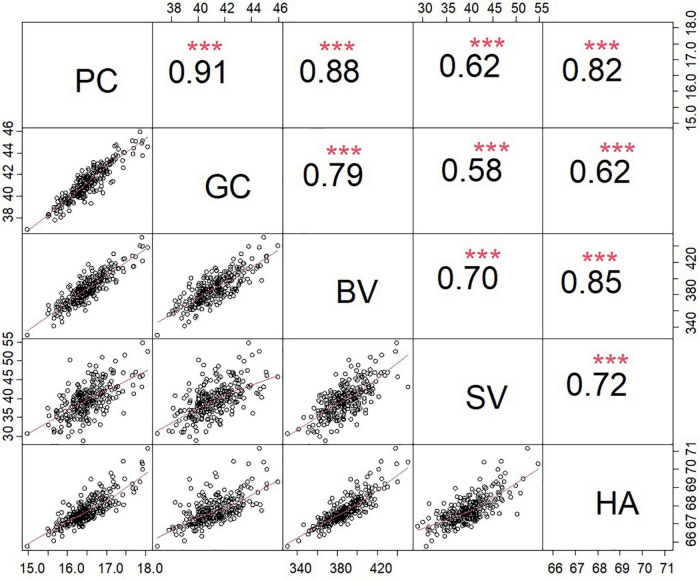
Correlations among five quality traits. ***Represents significance at 0.001 level.

The analysis of the association panel revealed considerable variations in all traits evaluated across all environments (Supplementary Table 1). Descriptive statistics for these traits are presented in Table 2. The variations ranged from 14.34 to 19.68% (16.51% ± 0.47%) for PC, 34.54% to 51.53% (41.49% ± 1.51%) for GC, 294.20 to 517.87 (387.30 ± 18.18) for BV, 21.13 to 68.78 mm (39.63 ± 4.26 mm) for SV, and 64.38 to 73.95 (67.76 ± 0.76) for HA. The combined variance analysis of the five traits detected significant differences between the genotypes of the association populations, the environments, and the genotype × environment interactions. The heritability (*h*^2^) of the five traits ranged from 73.13% to 76.96% (Table 2). Significant positive correlations were detected among the five traits according to the phenotypic BLUP values, with the correlation coefficient ranging from 0.80 (between PC and GC) to 0.91 (between GC and SV) (Figure 2).

**TABLE 2:** Descriptive statistics, broad-sense heritability, and analysis of variance for the five quality traits in the association panel.

Trait	Environment	Mean	SD	CV%	Min	Max	Range	*h* ^2^	F-values from ANOVA	
									Genotype	Environment
PC	HJHQ	16.51	0.71	4.31	14.51	19.56	5.05	76.09	3.86***	14.85***
	LH	16.38	0.77	4.71	14.34	19.27	4.93			
	WLTQQ	16.49	0.88	5.29	14.39	19.68	5.29			
	BLUP	16.51	0.47	2.89	14.98	18.05	3.07			
GC	HJHQ	40.65	2.48	6.09	35.74	49.89	14.15	73.13	3.54***	16.05***
	LH	41.40	2.57	6.21	34.54	50.50	15.96			
	WLTQQ	41.54	2.81	6.78	35.12	51.53	16.41			
	BLUP	41.19	1.51	3.67	36.93	45.96	9.03			
BV	HJHQ	395.87	27.68	6.99	301.49	517.87	216.38	73.98	3.26***	201.84***
	LH	364.18	31.32	8.60	294.20	472.32	178.12			
	WLTQQ	401.36	31.37	7.82	318.45	503.83	185.38			
	BLUP	387.30	18.18	4.69	330.11	450.55	120.44			
SV	HJHQ	41.66	7.14	17.14	21.84	63.41	41.57	76.10	3.51***	347.91***
	LH	33.10	6.44	19.47	21.13	54.61	33.48			
	WLTQQ	44.36	7.05	15.92	28.44	68.78	40.34			
	BLUP	39.63	4.26	10.76	28.86	54.73	25.87			
HA	HJHQ	67.96	1.17	1.74	64.47	72.32	7.85	76.96	3.98***	168.22***
	LH	66.98	1.21	1.80	64.38	72.17	7.79			
	WLTQQ	68.34	1.32	1.93	65.37	73.95	8.58			
	BLUP	67.76	0.76	1.12	65.77	71.15	5.38			

***Represents significance at 0.001 level.

### Loci Significantly Associated With Five Quality Traits

**FIGURE 3:**
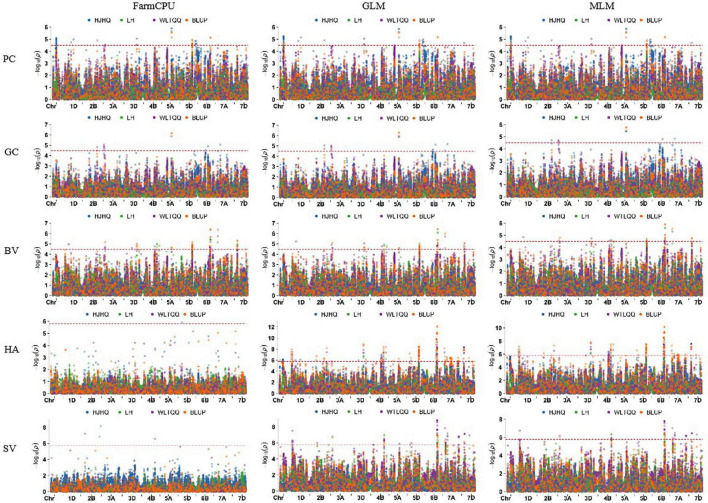
Manhattan plots for five quality traits analyzed using three models and BLUP values as well as data from individual environments.

Using 30,792 polymorphic SNPs (missing rate ≤ 10% and MAF ≥ 5%), a GWAS involving the GLM, MLM, and FarmCPU models was performed to analyze five quality traits. A total of 556 SNPs significantly associated with the five traits were detected on the basis of the phenotypic data for the different environments and the BLUP values (Supplementary Table 2, Figure 3, and Supplementary Figure 3). These SNPs were distributed over all wheat chromosomes, with the exception of chromosome 3A. Additionally, the SNPs were relatively concentrated in some chromosomal regions (e.g., the long arm of chromosomes 1A, 4B, 2D, 5D, and 6B). Among the examined traits, HA was associated with the most SNPs (260), which was followed by PC (108), SV (83), BV (70), and GC (35). On the basis of the average LD decay distance (about 4 Mb) in the genomes of the 253 accessions, significant SNPs within a 4-Mb region were combined as a single QTL. Thus, 103 QTL were identified for the five quality traits (Supplementary Table 3). The number of QTL per trait ranged from 15 for GC to 37 for HA. The 103 significant QTL were almost evenly distributed among the three subgenomes, with 32 on the A subgenome, 37 on the B subgenome, and 34 on the D subgenome. But they were unevenly distributed on different chromosomes, ranging from 1 (2D and 6B) to 11 (2A and 5B), indicating that the power of QTL identification was affected by marker density. Of these QTL, 32 were identified simultaneously in at least one environment and on the basis of BLUP values, including 2 for each for PC and GC, 8 for BV, 15 for HA and 5 for SV (Table 3).

**TABLE 3:** Details regarding the 32 stable QTL identified simultaneously in at least one environment and on the basis of BLUP values.

Trait	QTL	No. SNPs	Chr	Region (Mb)	Environments (Model)
PC	*qPC1A*	68	1A	301.16–318.19	HJHQ (FarmCPU/GLM/MLM), BLUP (GLM)
	*qPC5A.3*	3	5A	479.28–479.32	HJHQ (FarmCPU/GLM/MLM), BLUP (FarmCPU/GLM/MLM)
GC	*qGC2B.2*	2	2B	723.96–731.60	WLTQQ (FarmCPU/GLM/MLM), BLUP (FarmCPU)
	*qGC5A*	3	5A	479.28–479.32	HJHQ (FarmCPU/GLM/MLM), BLUP (FarmCPU/GLM/MLM)
BV	*qBV2D.2*	2	2D	481.59–489.72	WLTQQ (FarmCPU/GLM), BLUP (FarmCPU/GLM/MLM)
	*qBV3D.2*	3	3D	571.36–572.83	WLTQQ (FarmCPU/GLM), BLUP (FarmCPU/GLM)
	*qBV5D*	25	5D	547.50–549.49	LH (FarmCPU/GLM/MLM), BLUP (FarmCPU/GLM/MLM)
	*qBV6B.1*	12	6B	613.35–632.44	LH (FarmCPU/GLM/MLM), BLUP (FarmCPU/GLM)
	*qBV6B.2*	7	6B	658.73–662.45	LH (FarmCPU/GLM/MLM), WLTQQ (FarmCPU/GLM/MLM), BLUP (FarmCPU/GLM/MLM)
	*qBV6D*	1	6D	437.62	LH (FarmCPU/GLM), WLTQQ (FarmCPU/GLM/MLM), BLUP (FarmCPU/GLM/MLM)
	*qBV7B.1*	1	7B	605.96	WLTQQ (GLM/MLM), BLUP (FarmCPU/GLM)
	*qBV7B.2*	7	7B	634.30–635.02	LH (FarmCPU/GLM), WLTQQ (FarmCPU/GLM), BLUP (FarmCPU/GLM/MLM)
HA	*qHA1A.1*	1	1A	54.5	WLTQQ (GLM), BLUP (GLM)
	*qHA1B.1*	8	1B	336.20–343.25	WLTQQ (GLM/MLM), BLUP (GLM/MLM)
	*qHA2B.2*	1	2B	105.48	WLTQQ (GLM), BLUP (GLM/MLM)
	*qHA2D.1*	1	2D	34.23	WLTQQ (GLM/MLM), BLUP (GLM/MLM)
	*qHA3D.2*	3	3D	571.36–572.83	LH (FarmCPU/GLM), WLTQQ (FarmCPU/GLM/MLM), BLUP (FarmCPU/GLM/MLM)
	*qHA4B.1*	2	4B	465.74–465.90	WLTQQ (GLM), BLUP (GLM)
	*qHA4B.2*	29	4B	477.23–481.80	WLTQQ (GLM/MLM), BLUP (GLM)
	*qHA4B.3*	12	4B	658.66–659.16	LH (GLM/MLM), WLTQQ (GLM), BLUP (GLM/MLM)
	*qHA4D*	1	4D	47.32	LH (GLM/MLM), WLTQQ (GLM/MLM), BLUP (GLM/MLM)
	*qHA5D*	39	5D	547.50–550.44	LH (GLM/MLM), WLTQQ (GLM/MLM), BLUP (GLM/MLM)
	*qHA6B.2*	23	6B	613.35–632.44	HJHQ (GLM/MLM), LH (GLM/MLM), WLTQQ (GLM/MLM), BLUP (GLM/MLM)
	*qHA6B.3*	19	6B	653.06–662.45	HJHQ (GLM/MLM), LH (GLM/MLM), WLTQQ (GLM/MLM), BLUP (GLM/MLM)
	*qHA6D.1*	1	6D	437.62	WLTQQ (GLM/MLM), BLUP (GLM/MLM)
	*qHA7A.1*	3	7A	280.11–284.15	WLTQQ (GLM), BLUP (GLM/MLM)
	*qHA7B.2*	7	7B	605.96–635.02	WLTQQ (GLM/MLM), BLUP (GLM/MLM)
SV	*qSV2D.3*	1	2D	489.72	WLTQQ (GLM/MLM), BLUP (FarmCPU/GLM)
	*qSV4B*	13	4B	649.58–659.16	LH (GLM/MLM), WLTQQ (FarmCPU/GLM/MLM), BLUP (GLM)
	*qSV6B.1*	23	6B	613.35–632.44	LH (GLM), WLTQQ (GLM/MLM), BLUP (GLM/MLM)
	*qSV6D.1*	1	6D	437.62	WLTQQ (GLM/MLM), BLUP (GLM)
	*qSV6D.2*	11	6D	470.09–470.96	LH (GLM), WLTQQ (FarmCPU/GLM)

Interestingly, several QTL were associated with multiple quality traits. For example, the QTL on chromosome 5A (*qPC5A.3/qGC5A*; 479.28–479.32 Mb) were associated with PC and GC, whereas the QTL on chromosomes 3D (*qBV3D.2/qHA3D.2*; 571.36–572.83 Mb), 5D (*qBV5D/qHA5D*; 547.50–549.49 Mb), and 6D (*qBV6D/qHA6D.1*; 437.62 Mb) were associated with BV and HA. Additionally, two QTL on chromosome 6B (*qBV6B.1/qHA6B.2*; 613.35–632.44 Mb and *qBV6B.2/qHA6B.3*; 653.06–662.45 Mb) related to BV were also associated with HA and SV.

**FIGURE 4:**
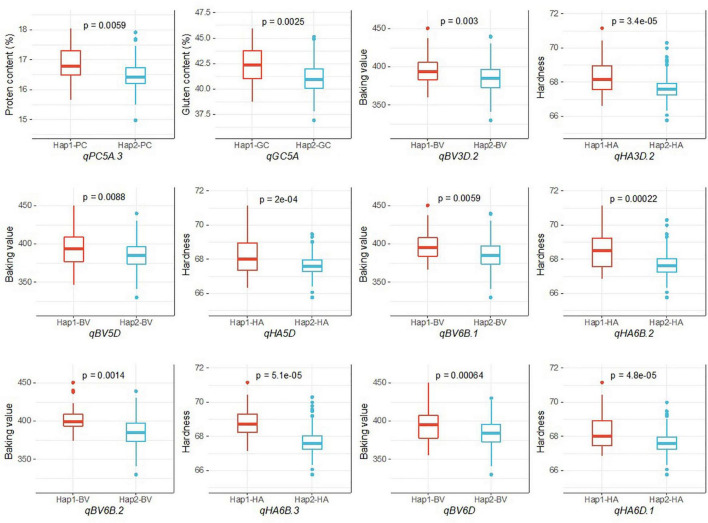
Analysis of the haplotype effects of the stable QTL on chromosomes 3D, 5A, 5D, 6B, and 6D.

Because stability across different environments and models is an important parameter for evaluating the reliability of QTL, during our haplotype analysis, we focused on the QTL on chromosomes 3D, 5A, 5D, 6B, and 6D that were detected in at least two environments by all three models and had pleiotropic effects on multiple traits. We divided 252 accessions into two haplotypes according to the allele types of the three SNPs in the common QTL for PC and GC on chromosome 5A. Hap1 had a higher average PC (16.88%) and GC (42.33%) than Hap2 (16.47% for PC and 41.06% for GC). The 252 accessions were also divided into two haplotypes according to the allele types of the SNPs in the five common QTL for BV and HA on chromosomes 3D, 5D, 6B, and 6D. There were significant differences in the phenotypic values between the haplotypes (Table 4 and Figure 4).

**TABLE 4:** Analysis of the haplotype effects of the stable QTL on chromosomes 3D, 5A, 5D, 6B, and 6D.

Trait	QTL	Haplotype	Allele	No. accession	BLUP
PC	*qPC5A.3*	Hap1-PC	GCC	24	16.88
		Hap2-PC	TGT	228	16.47
GC	*qGC5A*	Hap1-GC	GCC	24	42.33
		Hap2-GC	TGT	228	41.06
BV	*qBV3D.2*	Hap1-BV	CCC	47	394.96
		Hap2-BV	ATT	200	385.37
	*qBV5D*	Hap1-BV	CCG…CTT	55	393.84
		Hap2-BV	AGA…TGG	197	385.27
	*qBV6B.1*	Hap1-BV	TAC…CAA	28	397.5
		Hap2-BV	CGT…TGG	225	385.96
	*qBV6B.2*	Hap1-BV	GCTAATA	21	402.87
		Hap2-BV	TTCGCCG	232	385.83
	*qBV6D*	Hap1-BV	T	54	395.79
		Hap2-BV	C	198	384.78
HA	*qHA3D.2*	Hap1-HA	CCC	47	68.31
		Hap2-HA	ATT	200	67.63
	*qHA5D*	Hap1-HA	CCG…CTT	55	68.22
		Hap2-HA	AGA…TGG	197	67.62
	*qHA6B.2*	Hap1-HA	TAC…CAA	28	68.57
		Hap2-HA	CGT…TGG	225	67.66
	*qHA6B.3*	Hap1-HA	GCTAATA	21	68.81
		Hap2-HA	TTCGCCG	232	67.66
	*qHA6D.1*	Hap1-HA	T	54	68.25
		Hap2-HA	C	198	67.62

### Relationship Between the Quality Traits and the Number of Favorable Alleles

**FIGURE 5:**
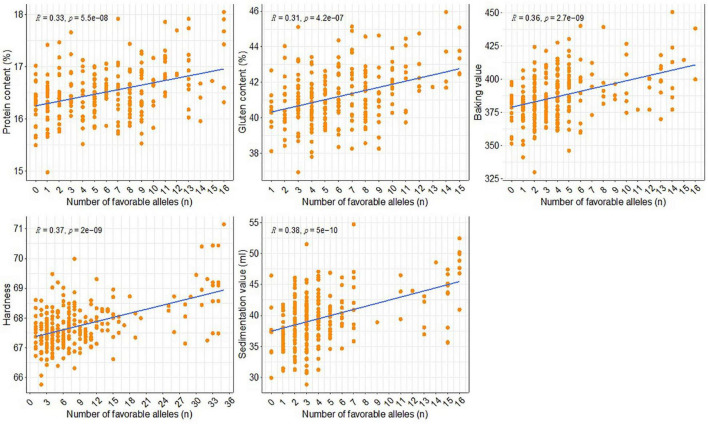
Distribution of the favorable alleles of the stable QTL in the association panel and their effects on five quality traits.

In this study, marker alleles with positive additive effects leading to increased phenotypic values were considered to be favorable alleles. Allelic effects were simulated for all five examined quality traits using the most significant SNPs in each QTL interval. The relationship between the effects of the favorable and unfavorable alleles was determined by analyzing the BLUP values for the phenotypes. The number of favorable alleles in individual accessions ranged from 0 to 16, 1 to 15, 0 to 16, 1 to 35, and 0 to 16 for PC, GC, BV, HA, and SV, respectively (Figure 5). The accessions were grouped according to the number of favorable alleles for each trait. A significant linear relationship was observed between the traits and the number of favorable alleles. In other words, increases in the number of favorable alleles were associated with increases in the phenotypic values for the traits. More specifically, as the number of favorable alleles increased, the values for PC, GC, BV, HA, and SV increased by 1.05%, 2.99%, 41.00, 3.60, and 10.85 ml, respectively (Figure 5).

### Identification and Functional Characterization of Candidate Genes

In this study, the 4-Mb sequences flanking the significant SNPs in each stable QTL associated with five quality traits were regarded as potential candidate gene regions. A total of 4,176 putative candidate genes were identified by screening the annotated genes in the “Chinese Spring” reference genome database (IWGSC RefSeq v1.1) (Supplementary Table 4). To further speculate on the causal genes responsible for the formation of wheat quality, we analyzed the expression characteristics of these genes in different developmental stages and tissues using RNA-seq data available in an online wheat gene expression database (see text footnote 4). It was found that 34 genes (16 of those were associated with at least two traits) were specifically and highly expressed in grains at the reproductive growth stage, from 4 for PC to 23 for HA (Supplementary Table 5 and Supplementary Figure 4). These genes were predicted to encode proteins with various functions, such as aquaporin (*TraesCS1A02G165400*), vacuolar-processing enzyme (*TraesCS2D02G382400*, *TraesCS2D02G383400*, *TraesCS2D02G383500*), cytochrome P450 (*TraesCS5D02G535400*), zinc finger CCCH domain-containing protein (*TraesCS2B02G138100*), phosphoglycerate kinase (*TraesCS2D02G081700*), divalent metal cation transporter (*TraesCS4B02G381200*), tropinone reductase-like protein (*TraesCS5D02G530100*), but also include some proteins with unknown functions (Supplementary Table 5). Given that wheat quality is closely related to grain development and its composition, these specifically expressed genes are most likely to be key causal genes for wheat quality traits, and their functions were worth further investigation in the future.

## Discussion

Wheat has been cultivated in China for more than 4,000 years, and it is now grown in 10 major agro-ecological regions (Hao et al., 2008; Sun et al., 2017). High-quality spring wheat is cultivated in the Hetao Irrigation District in Inner Mongolia because of the superior regional environmental conditions (e.g., light and heat) as well as the availability of water sources. However, the genetic diversity of the wheat in this region is low, resulting in varietal degradation and poor stress resistance. Clarifying the genetic basis of the quality traits in Hetao wheat varieties is important for ensuring the efficient and sustainable development and competitiveness of the Hetao wheat industry through the breeding of improved varieties. In this study, we evaluated five quality traits and the population structure in a panel comprising 253 wheat accessions. Additionally, we conducted a GWAS using a wheat 55 K SNP array to analyze wheat grown in multiple environments in Inner Mongolia.

Wheat quality is a complex and comprehensive trait influenced by individual traits and their interrelationships and interactions. In the current study, strong correlations between individual traits were revealed by the BLUP data (Figure 2), which are consistent with those of other recent studies (Gao et al., 2021; Lou et al., 2021). Additionally, significant variations in individual traits were observed in the 253 accessions included in this study. However, few materials (Longmai 15–5049) performed well in multiple traits at the same time. In this regard, there is an urgent need to screen or introduce more excellent parents, which is crucial for the continuous cultivation of high-quality wheat with excellent comprehensive traits.

The LD within a population forms the basis of GWAS, but it is often influenced by mutations, genomic rearrangements, genetic draft, population stratification, and natural selection (Al-Maskri et al., 2012). Population stratification is considered to be the major factor affecting the accuracy of GWAS results (Cardon and Palmer, 2003). In the current study, the ADMIXTURE and principal component analysis produced the same results regarding the population structure, with the 253 accessions divided into four subgroups (Figure 1). Because wheat is a self-pollinating species with a very large genome, its LD decay distance is larger than that of other plants, including maize, rice, and Arabidopsis (Andersen et al., 2007; Kim et al., 2007; Mather et al., 2007; Al-Maskri et al., 2012). Moreover, LD decay can vary among the mapping populations of the same species. In this study, LD decay was greatest in the D genome, whereas Ain et al. (2015) and Sun et al. (2017) reported that LD decay was greatest in the A and B genomes, respectively.

LD decay distance is generally used to determine candidate intervals for the target QTL/genes. The LD decay distance shown in this study are similar to those reported in previous studies (Jin et al., 2020; Wu et al., 2021). It is important to note, however, this result should be interpreted with caution. Due to the large variation in LD between chromosomes and within chromosomes, and the diversity of methods for determining LD decay distance thresholds, the LD decay distance used to specify candidate intervals is only an approximation, and also that candidate genes in same cases may have not been captured within the identified intervals.

In our GWAS, 556 significant SNPs in 103 QTL were associated with the five examined quality traits. Because genetic analyses performed to identify the genomic regions corresponding to quantitative traits are often influenced by the genotype × environment interaction, stability across diverse environments is an important parameter for assessing the value and reliability of a particular locus. We focused on 32 QTL identified in at least two environments. These QTL are located on 12 of the 21 chromosomes in wheat (Table 3). We compared our results with those of previous investigations. For PC and GC, a common QTL (*qPC5A.3/qGC5A*) was detected on chromosome 5A; several QTL on this chromosome have been identified (Sun et al., 2008, 2016; Tsilo et al., 2010; Echeverry-Solarte et al., 2015; Tiwari et al., 2015; Li et al., 2016). Another two QTL (*QGpc.WY-1A.1/QWgc.WY-1A.1* and *QGpc.WY-5A.1/QWgc.WY-5A.2*) associated with PC and GC were detected by Li et al. (2011). These results reflect the high correlation between PC and GC. Li et al. (2011) also identified two QTL (*QWgc.WJ-2B.1a* and *QWgc.WJ-2B.1b*) for GC on chromosome 2B. In the present study, a predicted GC QTL (*qGC2B.2*) was detected between the SNPs *Affx-111472438* and *Affx-109061076* on chromosome 2B. However, as QTL mapping provides only limited resolution, further experiments were required to determine whether the genome regions on 2B associated with GC in the two studies were the same. Regarding BV, relatively few QTL/genes were revealed in earlier studies involving map-based cloning or GWAS. Therefore, the eight stable QTL detected in this study may provide useful insights for future investigations of the genetic basis of BV. For HA, in addition to the *Pina/Pinb* gene on chromosome 5D, dozens of QTL related to this trait were mapped in previous studies (Arbelbide and Bernardo, 2006; Ma et al., 2010; Heo and Sherman, 2013; Boehm et al., 2018; Sun et al., 2018; Kumar et al., 2019). Several of these QTL are within one LD of a QTL (*qHA1A.1*, *qHA1B.1*, *qHA5D*, and *qHA7A.1*) revealed in the current study. Notably, two pleiotropic loci (*qHA6B.2/qHA6B.3*) affecting HA and BV were detected on chromosome 6B in all tested environments, indicating they may be major loci. Moreover, we detected five stable QTL for SV on four chromosomes (2D, 4B, 6B, and 6D). To the best of our knowledge, there are no published reports describing these QTL. Furthermore, the QTL (*qSV6B.1*) on chromosome 6B contains up to 23 significant SNPs. Hence, it should be more thoroughly examined in future studies to identify potential causal genes.

There is substantial interest in identifying the candidate genes underlying specific traits relevant for plant breeding. To date, researchers have identified many QTL related to wheat quality traits, but there has been little research aimed at identifying putative candidate genes in these genomic regions, with a few exceptions (e.g., Uauy et al., 2006; Chen et al., 2019). The PC is significantly higher in tetraploid wheat cultivars containing *GPC-B1* than in cultivars containing mutant or missing alleles. *Glu-1*, *Glu-3* and *Gli* genes encoding high molecular weight glutenin subunit, low molecular weight glutenin subunit and gliadin, respectively, were confirmed to be directly involved in the formation of grain proteins and had a great impact on GPC and WGC (Plessis et al., 2013; Wang et al., 2018). *Pina* and *Pinb* gene, encoding the friabilin components puroindoline a and b, are widely known to be major determinants of grain texture in common wheat (Giroux and Morris, 1998; Wang et al., 2018). In this study, candidate genes were predicted in the genomic regions surrounding 32 major and stable QTL (Table 3 and Supplementary Table 4). Our analysis of existing transcriptome data of wheat indicated that 34 genes were specifically highly expressed in grains during reproductive growth (Supplementary Table 5 and Supplementary Figure 4). For example, *TraesCS2D02G382400*, *TraesCS2D02G383400*, and *TraesCS2D02G383500*, encoding vacuolar processing enzymes, were identified as candidate genes for BV and SV. In rice, Glup3/OsVPE1 (vacuole-processing enzyme) belongs to the cysteine protease family, which processes gluten precursors into acidic and basic subunits in protein storage vacuole (PSV), and thus plays a key role in gluten maturation (Wang et al., 2009; Kumamaru et al., 2010; Ueda et al., 2010). *TraesCS2B02G138100*, which is related to HA, was predicted to encode a Zinc finger CCCH domain-containing protein. In rice, the down-regulated expression of *OsGZF1* (Cys3His1, a zinc finger gene) by RNAi reportedly leads to increased grain nitrogen concentration in transgenic plants (Chen et al., 2014). However, as molecular mechanism of wheat quality formation is quite complex, the role of genes encoding unknown functional proteins and the actual functions of presumed candidate genes should be carefully verified in the future.

## Data Availability Statement

The datasets presented in this study can be found in online repositories. The names of the repository/repositories and accession number(s) can be found in the article/Supplementary Material.

## Author Contributions

MY and MX conceived and designed the research. SH, HL, and HW performed the experiments. JS, Baogerile, JT, SM, QP, LY, MW, and MG analyzed the data. SH, HL, NZ, and JD performed the statistical data analysis and visualizations. SH, HL, MY, and MX wrote the original draft. MY, MX, and DL reviewed and edited the manuscript. All authors read and approved the last version.

## Conflict of Interest

The authors declare that the research was conducted in the absence of any commercial or financial relationships that could be construed as a potential conflict of interest.

## Publisher’s Note

All claims expressed in this article are solely those of the authors and do not necessarily represent those of their affiliated organizations, or those of the publisher, the editors and the reviewers. Any product that may be evaluated in this article, or claim that may be made by its manufacturer, is not guaranteed or endorsed by the publisher.

## Footnotes

1
http://www.cgmb.com.cn

2
http://www.wheatgenome.org/

3
http://wheat.cau.edu.cn/TGT/

4
http://www.wheat-expression.com

## Supplementary Material

Supplementary Figure 1 Chromosomal distribution of the SNPs used for the GWAS.

Supplementary Figure 2 Average LD decay distance in the genomes of 253 wheat accessions.

Supplementary Figure 3 Quantile–quantile plots for five quality traits analyzed using three models and BLUP values as well as data from individual environments.

Supplementary Figure 4 Expression heatmap of 34 key candidate genes based on transcriptome data.

Supplementary Table 1 Phenotypic datas of five quality traits in 253 accessions for three environments and BLUP values.

Supplementary Table 2 Significant SNP for five quality traits in different environments identified by GWAS.

Supplementary Table 3 A total of 103 QTL composed of significant snp detected by GWAS.

Supplementary Table 4 Annotation genes within the 4 Mb region flanking of the significant SNPs included in stable QTL for five quality traits.

Supplementary Table 5 Candidate genes specifically and highly expressed in grain at reproductive growth stages based on available transcriptome data.
