# Supplementary figures and images for: Genome-Wide Association Study Reveals the Genetic Basis of Five Quality Traits in Chinese Wheat
Source: Front Plant Sci. 2022 Mar 3;13:835306. doi: 10.3389/fpls.2022.835306 (PMC8928432; doi:10.3389/fpls.2022.835306)

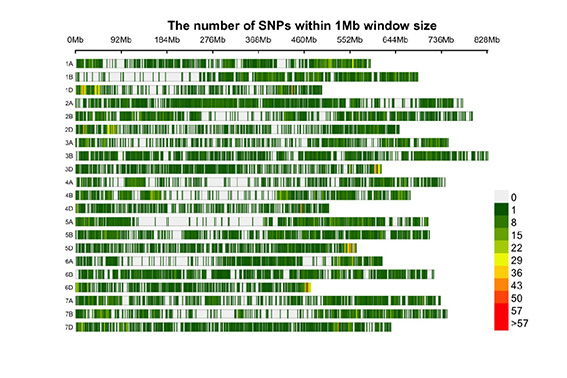

Supplement: Supplementary Figure 1 — Chromosomal distribution of the SNPs used for the GWAS. [file Image_1.JPEG]

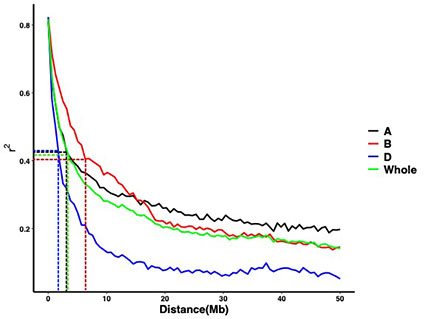

Supplement: Supplementary Figure 2 — Average LD decay distance in the genomes of 253 wheat accessions. [file Image_2.JPEG]

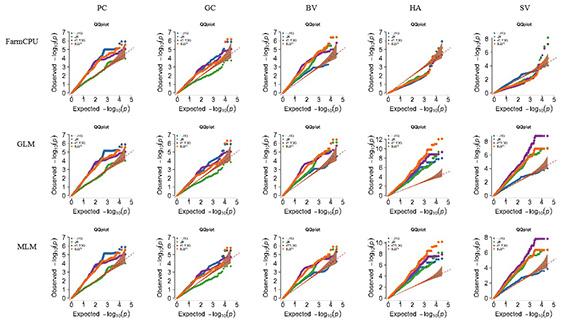

Supplement: Supplementary Figure 3 — Quantile–quantile plots for five quality traits analyzed using three models and BLUP values as well as data from individual environments. [file Image_3.JPEG]

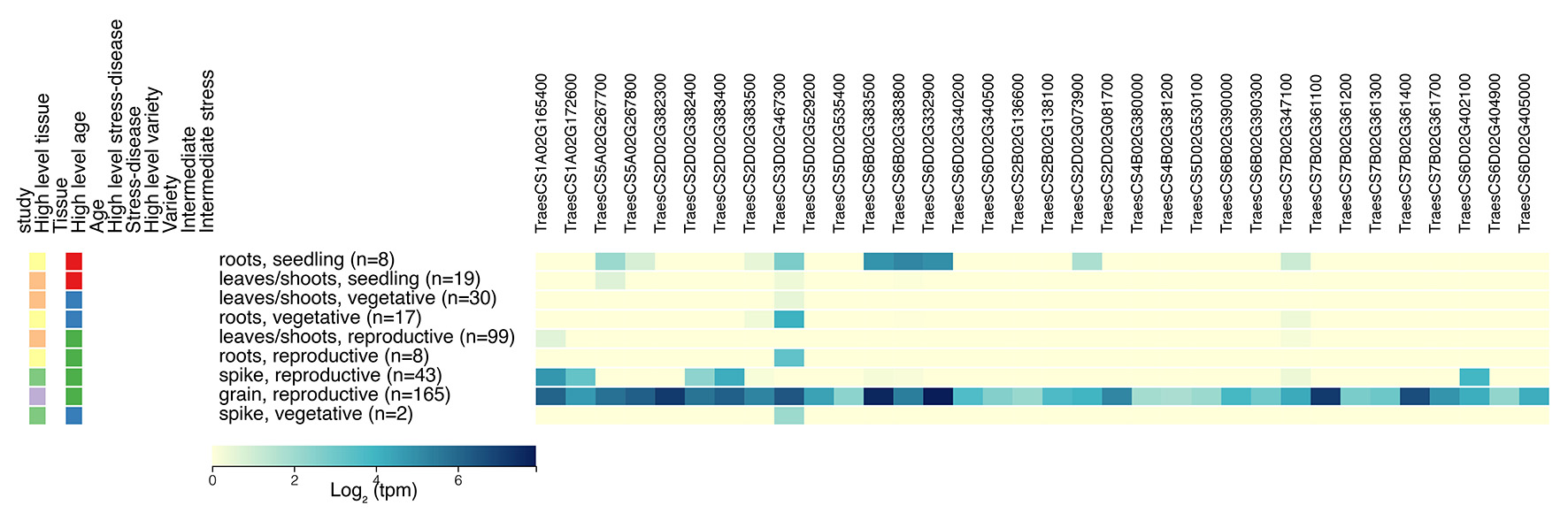

Supplement: Supplementary Figure 4 — Expression heatmap of 34 key candidate genes based on transcriptome data. [file Image_4.JPEG]
